# Research progress on the pharmacological effects of *Poria cocos*: a narrative update

**DOI:** 10.3389/fnut.2026.1774161

**Published:** 2026-01-29

**Authors:** Yanquan Guo, Tianyuan Liu, Dan Li

**Affiliations:** 1 Department of Pharmacy, Jining No.1 People’s Hospital, Jining, Shandong, China; 2 Yangzijiang Pharmaceutical Group, Taizhou, Jiangsu, China; 3 School of Pharmacy, Qilu Medical University, Zibo, Shandong, China

**Keywords:** active components, pachymic acid, pharmacological effects, *Poria cocos*, *Poria cocos* polysaccharides

## Abstract

*Poria cocos*, a traditional medicinal and food homologous herb, has various therapeutic effects, including promoting urination, dispersing dampness, strengthening the spleen, and calming the mind. Its core active ingredients mainly consist of polysaccharides and triterpenoids. Recent studies have shown that *Poria cocos* and its active components play a crucial regulatory role in various physiological and pathological processes. It demonstrates a wide range of pharmacological activities, including renal protection, improving digestive system function, enhancing sleep quality, immune regulation, antioxidant effects, anticancer properties, and the modulation of glucose-lipid metabolism. These mechanisms involve multifaceted regulation of gut microbiota and glycolipid metabolism. This review systematically summarizes the latest research progress on the chemical composition and pharmacological effects of *Poria cocos* over the past 3 years (2023–2025), aiming to provide a scientific basis for its further development and clinical application.

## 1 Introduction

*Poria cocos* is derived from the dried sclerotium of the basidiomycete fungus *Poria cocos* (Schw.) Wolf., a species that parasitizes the roots of pine trees such as red pine or masson pine. As a traditional medicinal material with a long history of use, it has attracted considerable attention for its distinctive pharmacological effects. More than two millennia ago, the *Shennong Bencao Jing* cited it as a paradigm of “medicine and food of the same origin,” noting its diuretic and dampness-eliminating effects, its ability to strengthen the spleen and harmonize the stomach, and its calming of the mind; in traditional medicine it has been widely used to treat edema, insomnia, and related conditions (1). With rapid advances in modern biomedicine, the pharmacological value of *Poria cocos* has been further explored, and its diverse roles in disease prevention and treatment are increasingly substantiated by scientific evidence, making it a focal point of modernized traditional medicine research. The pharmacological activities of *Poria cocos* are closely linked to its complex chemical composition, with polysaccharides and triterpenoids identified as core active constituents, and in recent years new bioactive constituents have continued to be isolated from *Poria cocos*. We searched for research literature related to *Poria cocos* in the PubMed, CNKI, and Web of Science databases. The search keywords were “*Poria cocos*” or “Fuling,” and the search period was from 2023 to 2025. Review articles and duplicate publications were excluded, and a review was conducted on the selected literature. In addition to summarizing active components, this review emphasizes the pharmacological actions and cutting-edge molecular mechanisms across multiple domains, including renal protection, regulation of the digestive system, sleep improvement, immune modulation, antioxidant activity, anti-tumor effects, and regulation of carbohydrate and lipid metabolism. The aim is to enable researchers to quickly grasp the latest research directions of *Poria cocos* and provide comprehensive literature references and scientific basis for the in-depth development and utilization of *Poria cocos*, a resource that is both medicinal and edible.

## 2 Research on the main chemical components of *Poria cocos*

### 2.1 *Poria cocos* polysaccharides

*Poria cocos* polysaccharides (PCP) are predominantly present in the sclerotium and constitute one of the principal bioactive constituents. Their chemical structures are complex, comprising a main chain and side chains. Variation in chain length, degree of branching, and monosaccharide composition underpins the heterogeneity among PCP. Gu et al. (2) isolated and purified a water-soluble polysaccharide of 20.112 kDa from *Poria cocos*, predominantly composed of →6)-*α*-D-Galp-(1→, with minor amounts of →3)-*β*-D-Glcp-(1 → and →4)-β-D-Glcp-(1→ Lv et al. (3) isolated a 2.35 kDa water-soluble polysaccharide, PCP-2, composed of glucose, galactose, mannose, and fucose; its main-chain types include a glucan backbone with 1,3-β-D-Glc and 1,6-β-D-Glc linkages and a galactan backbone with 1,6-α-D-Gal units. Zhai et al. (4) employed a deep eutectic solvent to extract PCP-1 (MW 3.2 kDa) from *Poria cocos*, with a main glycosidic linkage of 1,3-linked Glcp; it adopts a triple-helix conformation, with β-D-Glcp-(1 → linked to the main chain via the O-6 atom to form the backbone. Sun et al. (5) isolated PCP-W1 with a molecular weight of 18.38 kDa, comprising galactose, glucose, fucose, and mannose in molar percentages of 35.87:28.56:21.77:13.64, displaying irregular coiling and branched conformational features. Li et al. (6) isolated four polysaccharide components from the culture filtrate and mycelia of *Poria cocos*, namely EPS-0M, EPS-0.1M, IPS-0M, and IPS-0.1M; these are composed of five monosaccharides-glucose, mannose, galactose, fucose, and rhamnose-in varying molar ratios.

### 2.2 Triterpenoids

Triterpenoids constitute another important class of bioactive constituents in *Poria cocos*, with the triterpenoids predominantly comprising lanostane-type tetracyclic triterpenes. These compounds display substantial structural diversity, with Lu et al. (7) having catalogued more than 100 triterpenoids reported in *Poria cocos*. In recent years, new triterpenoids from *Poria cocos* have been progressively identified. Wang et al. (8) isolated and characterized three new lanostane-type triterpenoids from *Poria cocos*, namely 12-hydroxydehydrotumulosic acid, tuckahoacid V, and tuckahoacid W. Bao et al. (9) isolated 17 new lanostane-type triterpenoids, tuckahoacid A-Q, from the *Poria cocos*. Xu et al. (10) isolated two new triterpenoids from the *Poria cocos*, namely (20R)-16α,24,31-trihydroxy-3,4-seco-lanosta-4(28),7,9(11)-triene-3,21-dioic acid 3-oate and (20R)-3β,23-dihydroxy-lanosta-7,9(11),24(25)-triene-21-oic acid.

## 3 Pharmacological effects of *Poria cocos*

### 3.1 Renal protective effects

The effects of *Poria cocos* on the kidneys are dependent on its diuretic and damp-dispelling properties. The study has shown that carboxymethylated *Poria cocos* polysaccharides (CMP) (200 mg/kg) could reduce the levels of inflammatory factors, such as tumor necrosis factor-alpha (TNF-α), interleukin-6 (IL-6), and IL-1β, in the peripheral blood and kidney tissues of septic rats with acute kidney injury. It also increases the expression levels of NAD(P)H quinone dehydrogenase 1, heme oxygenase-1, and glutathione peroxidase 4 (GPX4) in kidney tissues, stimulates nuclear factor erythroid 2-related factor 2 (Nrf2) activation to promote antioxidant gene expression, and inhibits the activation of the nuclear factor-kappa B (NF-κB) signaling pathway, thus protecting the kidneys (11). Water-soluble *Poria cocos* polysaccharides (WPCP), as the main active component of *Poria cocos*, have been shown, both *in vitro* (200 mg/kg) and *in vivo* (1 mg/mL), to offer significant protection against lipopolysaccharide (LPS)-induced septic acute kidney injury. The key mechanism is the inhibition of IκBα phosphorylation, which blocks NF-κB activation and subsequently suppresses nicotinamide adenine dinucleotide phosphate (NADPH) oxidase activation and NADPH oxidase 4 expression, exerting anti-inflammatory, antioxidant, and anti-apoptotic effects (12). Wang et al. (13) found that *Poria cocos* extract (PCE) (2 g/kg and 4 g/kg) reduces serum uric acid, creatinine, and urea nitrogen levels in hyperuricemic rats. It selectively regulates xanthine dehydrogenase and fatty acid synthase, affecting their metabolic pathways and alleviating kidney damage. The active component of *Poria cocos*, pachymic acid (PA) (25 mg/kg and 50 mg/kg), has a significant protective effect on fructose-induced hyperuricemic nephropathy in mice, improving renal dysfunction as well as pathological damage, such as renal tubular dilation and glomerular fibrosis. PA could directly bind to Nrf2 and promote its nuclear translocation, activating the Nrf2/GPX4 signaling pathway. This subsequently inhibits kidney iron accumulation, lipid peroxidation, and ferroptosis, while also alleviating oxidative stress and inflammatory responses (14).

Poricoic acid A (PAA) (20 mg/kg) has protective effects on kidney fibrosis induced by a high-salt diet in mice. It could reduce kidney index and urinary protein levels, improve renal tubular dilation and kidney tissue fibrosis, and activate the AMP-activated protein kinase (AMPK) signaling pathway in the kidneys. The core mechanism involves regulating the balance of the gut microbiota, increasing the abundance of beneficial bacteria such as *Lactobacilli* and *Akkermansia*, reducing the proportion of endotoxin-producing bacteria like *Desulfovibrio*, and promoting the production of short-chain fatty acids (SCFAs) such as acetate, propionate, and butyrate (15). PAA (10 mg/kg) improved kidney fibrosis induced by unilateral ureteral obstruction in mice. It alleviates pathological damage in kidney tissues, collagen deposition, and the epithelial-mesenchymal transition (EMT) process. The mechanism involves inhibiting endoplasmic reticulum stress (ERS) activation, downregulating the expression of proteins associated with the PERK-eIF2α-ATF4-CHOP signaling pathway (including glucose-regulated protein 78, activating transcription factor 4, and C/EBP homologous protein), and alleviating ERS-mediated renal tubular epithelial cell apoptosis (16, 17). Similarly, PAA inhibits transforming growth factor-beta 1 -induced EMT in renal tubular epithelial cells, reducing the expression of fibrosis markers such as *α*-SMA and collagen while increasing E-cadherin levels. It also suppresses cell proliferation, migration, and invasion. PAA (10 μM) could directly bind to the Sprouty RTK Signaling Antagonist 2 (SPRY2) protein and enhance its stability through the ubiquitin-proteasome pathway, leading to the downregulation of extracellular signal-regulated kinase (ERK) phosphorylation. Silencing SPRY2 could reverse the protective effects of PAA as described above (18). Furthermore, *Poria cocos* alcohol extract (0.6 g/kg) reduces serum atrial natriuretic peptide and IL-6 levels in rats with nephrogenic edema, demonstrating its beneficial effects on nephrogenic edema (19). PAA (100 μg/mL) exhibits significant protective effects against high-glucose-induced MPC5 podocyte injury. It promotes cell proliferation and migration, enhances autophagy, and inhibits apoptosis, inflammation, and oxidative stress. PAA activates AMPKα phosphorylation, which in turn inhibits FUN14 domain containing 1 (FUNDC1) phosphorylation. It regulates podocyte mitochondrial membrane potential and autophagy-related protein expression (such as microtubule-associated protein 1 light chain 3 and autophagy related 5) through the AMPKα/FUNDC1 pathway (20).

### 3.2 Effects on the digestive system

The effects of *Poria cocos* on the digestive system are related to its function in strengthening the spleen and stomach. *Poria cocos* aqueous extract (120 mg/kg) could reduce fecal moisture content in spleen deficiency model mice, increase the expression of Claudin and Occludin proteins, and slow down the small intestinal propulsion rate (21). Yang et al. (22) studied the effects of PCP (1.0 mg/mL) on patients with irritable bowel syndrome and found that the polysaccharides effectively regulated the abundance of gut microbiota, including *Lachnospiraceae* and *Prevotella*. *Poria cocos* oligosaccharides (200 mg/kg) could improve spleen index and colon length in colitis model mice, inhibit pro-inflammatory factors such as TNF-α, IL-1β, and IL-6, promote the expression of mucins and tight junction proteins to protect the intestinal barrier, and selectively regulate the abundance of gut microbiota, including *Odoribacter*, *Muribaculum*, and *Desulfovibrio* (23). Similarly, PCP reversed ulcerative colitis by reducing inflammatory signaling activation and restoring the expression of tight junction proteins (24, 25). PCP (250 mg/kg) could effectively alleviate symptoms of antibiotic-associated diarrhea and improve pathological damage to the intestinal mucosa. PCP could upregulate the expression of colonic tight junction protein ZO-1 and its mRNA to enhance the intestinal mucosal barrier function. It also increases the richness and diversity of the gut microbiota, regulating the structure of *Parabacteroides_distasonis* and *Akkermansia_muciniphila*, as well as the abundance of seven specific bacterial species. The mechanism of action is related to the regulation of mRNA expression of genes such as forkhead box P3 and G protein-coupled receptor 41, which are involved in immune modulation and metabolic regulation (26).

The water-insoluble polysaccharides of *Poria cocos* (300 mg/kg) have potential prebiotic functions. They could alleviate antibiotic-associated diarrhea, restore intestinal barrier function, reduce the levels of inflammatory factors, and regulate the gut microbiota structure, including norank_f_*Muribaculaceae* (27). PCP (75 mg/kg, 150 mg/kg, and 300 mg/kg), with a molecular weight of 11.583 kDa, improve intestinal mucosal function and maintain intestinal homeostasis by upregulating the expression of proteins such as Occludin and mucin 2, modulating cytokine levels, improving gut microbiota structure, and increasing SCFAs levels. This effect is associated with the activation of the Wnt/β-catenin pathway (28). PCP could be effectively utilized by the gut microbiota in an *in vitro* fecal fermentation model. The core effect is to reshape the gut microbiota structure, increasing the abundance of beneficial bacteria such as *Lactobacillus* and *Bifidobacterium*, reducing the proportion of pathogenic bacteria such as *Escherichia-Shigella* and *Bilophila*, and lowering the pH of the fermentation system, enhancing the production of SCFAs such as acetate and propionate. Additionally, 1% (w/v) PCP could upregulate beneficial metabolites such as L-cysteine and dipeptides, downregulate harmful metabolites like xanthine, and there is a close correlation between microbiota changes and metabolite adjustments (29).

### 3.3 Effects on sleep improvement

The sleep-improving effects of *Poria cocos* are related to its calming and tranquilizing properties. Clinical studies have reported that PCE, when administered at a dose of 800 mg per night, significantly improves the overall sleep duration and reduces wake time during sleep in patients with sleep disorders (30). Hao et al. (31) found that after 4 weeks of continuous supplementation with *Poria cocos* (10 mL/day), patients with sleep disorders showed a 12.96% increase in total sleep time, a 59.94% decrease in the Pittsburgh sleep quality index score, and significant improvement in sleep quality.

### 3.4 Immunomodulatory effects

Modern pharmacological studies indicate that PCP are the primary active components responsible for modulating the immune system. PCP (200 mg/mL) could increase the levels of immunoglobulin M (IgM), IgG, and IgA in diarrhea model mice and enhance the activity of peritoneal macrophages (32). Water-soluble *Poria cocos* polysaccharide PCP-2 (100, 200, 400 mg/kg) could promote the development of immune organs, such as the thymus and spleen, in mice. It also increases the levels of IgG and IgA in the blood and enhances intestinal immune function (3). Treatment with PCP (800 μg/mL) could regulate the expression of immune-related genes in macrophages and reduce the secretion of TNF-*α* (33). *Poria cocos* polysaccharide PCP-W1 (400 μg/mL) could activate the release of nitric oxide (NO), IL-6, IL-β, TNF-α, CD86, and reactive oxygen species (ROS). It induces polarization of RAW 264.7 macrophages to the M1 type by regulating the TLR4/MD2/NF-κB pathway (5, 34).

### 3.5 Antioxidant effects

In recent years, the antioxidant effects of *Poria cocos* have been extensively studied. A study comparing the extracellular metabolites of eight *Poria cocos* strains and their *in vitro* antioxidant activities found that the GTR2 strain had the highest levels of extracellular flavonoids and total phenolic compounds, and the highest DPPH radical scavenging activity, indicating its superior antioxidant activity (35). Other studies have indicated that CMP (500 μg/mL) can inhibit the proliferation of HepG-2 cells and scavenge DPPH free radicals (36). After CMP was treated with *γ*-rays, the polysaccharide chains underwent depolymerization, but its antioxidant activity was enhanced (37). In antioxidant experiments using *Caenorhabditis elegans*, PCP (40 μg/mL) increased the nuclear localization of the skn-1 transcription factor, upregulated the transcription levels of glutathione S-transferase 4 (gst-4) and gst-7, reduced intracellular ROS levels, and enhanced antioxidant stress resistance (38). PCP (3.99 mg/mL) prepared via microbial degradation showed a concentration-dependent scavenging ability against DPPH, hydroxyl radicals, and superoxide anion radicals (39).

### 3.6 Antitumor effects

The study has found that *Poria cocos* inhibits gastric cancer. *In vitro* (100 μM) experiments, it has been shown to suppress the proliferation, invasion, and migration of gastric cancer cells, and reverse TNF-β-induced EMT. Its core mechanism involves inducing ferroptosis in gastric cancer cells, which is manifested by the downregulation of SLC7A11 and GPX4 expression, an increase in intracellular ROS levels, and these effects could be reversed by the ferroptosis inhibitor Fer-1 (40). PA (20 μM) and *Poria cocos* ethanol extract (PCEE) (200 μg/mL) could inhibit the proliferation of mouse gastric cancer cells through the phosphoinositide 3-kinase / protein kinase B (PI3K/AKT) pathway (41, 42). It has also been reported that PCP (200 mg/kg) could regulate the gut microbiota, enhance intestinal barrier function, and inhibit inflammatory pathways to prevent and treat colon cancer in mice (43). PAA (200 μg/mL) has significant inhibitory effects on lung cancer cells such as H460 and H1299. It can induce apoptosis and block the cell cycle at the G2/M phase, while exhibiting low toxicity to normal lung cells. Its mechanism involves directly targeting MEK1/2, inhibiting the activation of the MEK/ERK signaling pathway, and downregulating the expression of cell proliferation-related proteins. This effect can be reversed by the MEK activator C16-PAF (44). PA (60 μM) also exerts significant inhibitory effects on kidney cancer cells. It inhibits cell proliferation, migration, and invasion in a concentration-dependent manner and induces apoptosis, while showing low toxicity to normal kidney cells. The mechanism involves inhibiting the PI3K/AKT/NF-κB signaling pathway, downregulating the phosphorylation levels of pathway-related proteins, and modulating the EMT/MMP signaling pathway. This leads to a decrease in the expression of metastasis-promoting proteins such as Vimentin and MMP-2/9, while increasing the expression of epithelial markers like E-cadherin. *In vivo* experiments using the 786-O cell xenograft model confirmed that PA inhibits tumor growth. This effect can be reversed by the PI3K activator 740Y-P and enhanced by the PI3K inhibitor LY294002 (45).

### 3.7 Effects on diabetes improvement

The regulation of glucose metabolism by *Poria cocos* is beneficial for the treatment of diabetes. The study has shown that PCE (0.15 mL/10 g) could reduce blood lipid and glucose levels in mice fed a high-fat diet (HFD), improving fat distribution, glucose-lipid metabolism, and energy metabolism (46). Wu et al. (47) reported that PAA (20 mg/kg) could inhibit apoptosis and inflammation in a streptozotocin-induced diabetic nephropathy mouse model, reduce ROS production, and significantly lower blood glucose and urinary protein levels. Additionally, *Poria cocos* aqueous extract (equivalent to 18.75 g/kg herb) has been reported to potentially treat diabetes by protecting intestinal barrier function, inhibiting the NF-κB/ NLR family pyrin domain containing 3 (NLRP3) signaling pathway, and improving gut microbiota dysbiosis (48). *Poria cocos* triterpenoid extract (100 mg/L) is the main active component for treating diabetic ulcers, containing 56 compounds including PA and PAA. Its mechanism of action involves activating the PI3K-AKT signaling pathway, promoting the migration of human umbilical vein endothelial cells and the expression of CD31 and vascular endothelial growth factor to enhance angiogenesis, while inhibiting the release of inflammatory factors such as IL-1β and IL-6 in THP-1 cells and inducing M2 macrophage polarization (49).

### 3.8 Effects on lipid metabolism and liver injury regulation

*Poria cocos* has lipid-lowering effects and can regulate lipid metabolism to treat liver diseases. PCP (50 mg/kg) could regulate the gut microbiota structure in HFD-induced obese mice, increase the production of SCFAs, and subsequently activate the FGF21/PI3K/AKT signaling pathway. PCP could reduce body weight and fat accumulation, regulate glucose-lipid metabolism and intestinal barrier function, and alleviate obesity-related metabolic disorders through the gut microbiota-SCFAs-signaling axis (50). PCE has been confirmed in both *in vivo* (300 mg/kg) and *in vitro* (100 μg/mL) experiments to improve hyperlipidemia and reduce lipid accumulation in hepatocytes. The mechanism involves activating the peroxisome proliferator-activated receptor alpha (PPARα) pathway, downregulating the expression of genes involved in fatty acid synthesis (sterol regulatory element-binding protein 1, acetyl-CoA carboxylase 1, fatty acid synthase), and upregulating genes involved in cholesterol metabolism (liver X receptor alpha, cholesterol 7α-hydroxylase, low-density lipoprotein receptor) (51).

**Figure 1:**
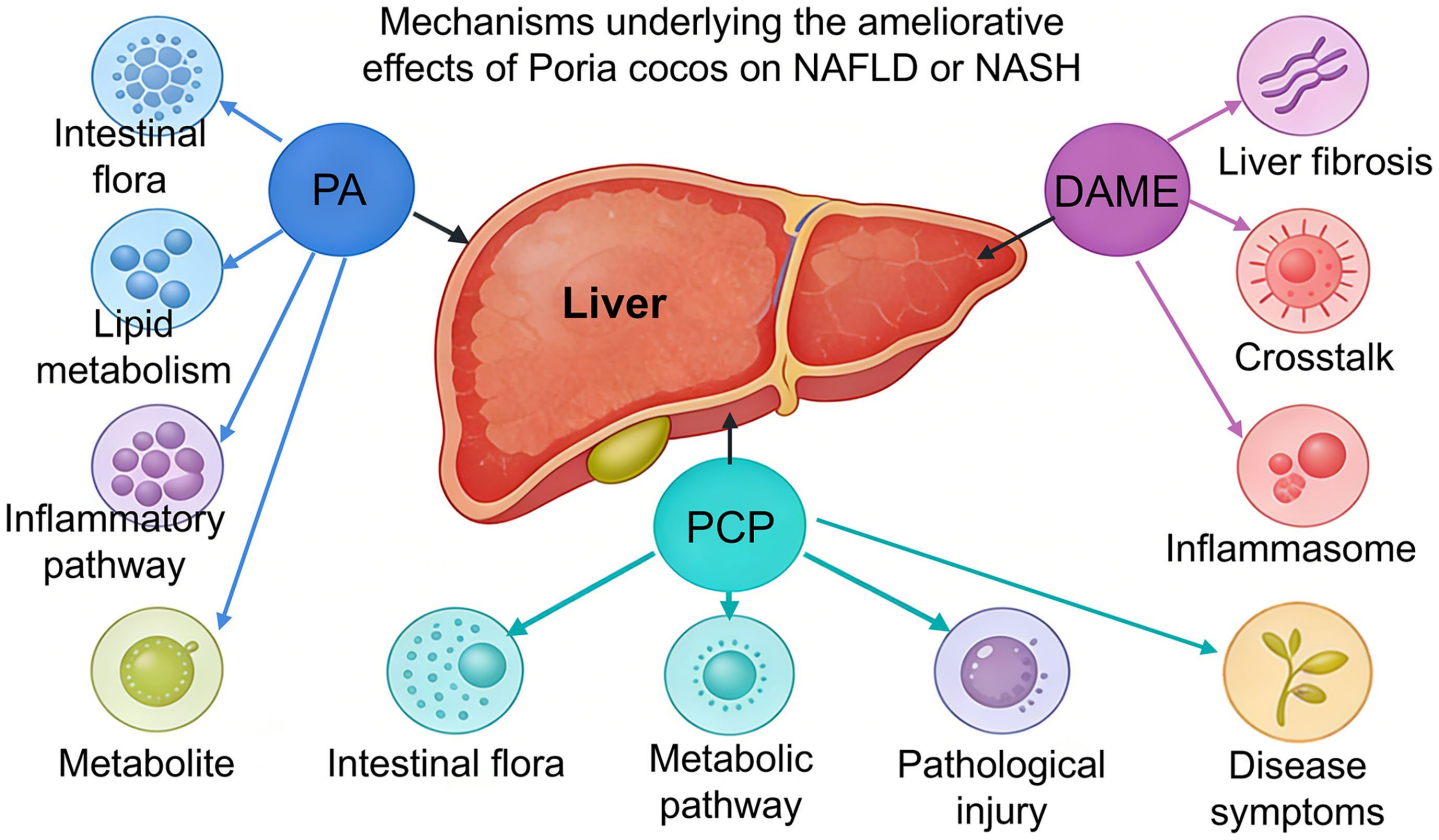
The active components in *Poria cocos* (including PA, PCP, and DAME) could ameliorate NAFLD/NASH through various pathways, such as modulating inflammatory responses, remodeling the gut microbiota, regulating lipid/metabolic pathways, and inhibiting liver fibrosis.

PA (40 mg/kg) could effectively alleviate HFD-induced non-alcoholic fatty liver disease (NAFLD), reducing hepatic lipid deposition, inflammation, and hepatocyte apoptosis. Its mechanism includes remodeling the gut microbiota balance (increasing *Akkermansia* abundance, decreasing *Desulfovibrio* and *Streptococcus* abundance, and improving the *Firmicutes/Bacteroidetes* ratio), regulating lipid metabolism pathways (inhibiting fatty acid synthesis genes such as FASN and SREBP1c, and upregulating lipid degradation genes such as PPARα and CPT1α), and alleviating liver inflammation through inhibition of the LPS/TLR4/MYD88/NF-κB pathway (52). Multi-omics analysis shows that PA could also regulate hepatic metabolite levels (e.g., increasing acylcarnitine and oleic acid), and changes in gut microbiota are closely associated with liver metabolism and inflammatory markers (52). Dehydrotrametenolic acid methyl ester (DAME) (75 mg/kg) improves non-alcoholic steatohepatitis (NASH) induced by HFD + CCl_4_ and Gubra-Amylin NASH (GAN) diet in ob/ob mice, alleviating liver damage, inflammatory infiltration, and liver fibrosis. DAME directly binds to the H236 site of mouse Caspase-1 (H237 site of human Caspase-1), inhibiting NLRP3 inflammasome activation, reducing the release of inflammatory factors such as IL-1β and IL-18, and regulating the crosstalk between macrophages, hepatocytes, and hepatic stellate cells (HSC), thereby alleviating hepatocyte lipid accumulation, apoptosis, and HSC activation. DAME also directly inhibits NLRP3 inflammasome activation in HSCs, downregulating the expression of fibrosis markers (53). PCP (236 mg/kg) improve NASH induced by a western diet combined with low-dose CCl_4_ in mice. They lower blood glucose and hepatic lipid levels, reduce liver inflammation, ballooning, and fibrosis, and improve fatigue-like behavior. PCP can reshape the gut microbiota structure, promoting the proliferation of beneficial bacteria such as *Alistipes* and *Butyricoccaceae_UCG-009*, while inhibiting harmful bacteria like *Romboutsia ilealis*. It also regulates hepatic metabolism pathways (especially taurine and taurocholic acid metabolism), restoring key metabolites such as tauroursodeoxycholic acid (54) (Figure 1). WPCP (100 mg/kg) alleviate alcoholic liver disease, alleviating liver damage, lipid accumulation, and inflammation, while enhancing intestinal barrier integrity. WPCP can reshape the gut microbiota (enriching *Parabacteroides distasonis*), upregulate intestinal levels of chenodeoxycholic acid and cholic acid, and activate the intestinal farnesoid X receptor/fibroblast growth factor 15 (FXR/FGF15) axis. This inhibits hepatic cholesterol 7α-hydroxylase to maintain bile acid homeostasis (55). PA (40 mg/kg) significantly alleviated metabolic-associated fatty liver disease induced by a HFD in mice. It lowers serum aspartate aminotransferase, alanine aminotransferase, and liver tissue TG and TC levels, reducing hepatocyte lipid accumulation and inflammatory infiltration. PA activates the PPARα, upregulates ferroptosis inhibitors such as GPX4 and SLC7A11, and downregulates the phosphorylation levels of transferrin receptor 1 and mitogen-activated protein kinase signaling pathway-related proteins. This reduces intracellular Fe^2+^ accumulation and lipid peroxidation in hepatocytes (56).

### 3.9 Others

PCE has a significant alleviating effect on hemorrhagic transformation (HT) after ischemic stroke induced by tissue-type plasminogen activator (tPA). PCE (84 mg/kg) regulates the interferon regulatory factor 5 (IRF5)-IRF4 axis, inhibiting M1 microglial polarization and promoting M2 polarization, reducing pro-inflammatory cytokine secretion and increasing anti-inflammatory cytokine release. *In vivo* and *in vitro* experiments confirmed that PCE reduces the hemorrhage and infarction volumes induced by tPA (57).

PCP improve cognitive impairment in 3 × Tg- Alzheimer’s disease (AD) mice, reducing Aβ deposition and neuronal damage in the brain. PCP (200 mg/kg) remodels the gut microbiota balance (increasing *Firmicutes* abundance and regulating dominant genera), elevating the levels of SCFAs like propionate and butyrate, thus repairing intestinal barrier integrity and blood–brain barrier function, while lowering peripheral LPS levels. At the same time, PCP alleviates neuroinflammation by inhibiting the TLR4/NF-κB signaling pathway, reducing CD8+ T cell infiltration in the brain, and blocking the microbiota-gut-brain axis-mediated pathological damage (58).

PCEE, containing active components such as PA and Tomoracic acid, can improve neurocellular cytoskeletal disorder and hyperactivity in mice induced by N-methyl-D-aspartate (NMDA) receptor antagonist MK-801. PCEE (10 mg/kg) regulates the Rho signaling pathway, restoring the expression and phosphorylation balance of proteins such as RhoA, CDC42, and Rac1, modulating the ROCK1-MLC2-PFN1 axis, and repairing F-actin polymerization and cell migration ability. *In vivo*, PCEE can reverse the abnormal Rho signaling in the prefrontal cortex of MK-801-treated mice, reducing locomotor hyperactivity (59).

*Poria cocos* oligosaccharides (PCO) (1 mg/mL) have significant protective effects on LPS-induced acute lung injury (ALI) in mice, reducing lung tissue pathology, inflammatory cell infiltration, and pulmonary edema. The mechanism includes inhibiting the NF-κB/NLRP3 signaling pathway to reduce the release of inflammatory factors such as IL-6 and TNF-*α*, upregulating the expression of aquaporin 5 and epithelial sodium channel to reverse pulmonary edema, while regulating plasma metabolic dysregulation. Metabolomics analysis shows that PCO can reverse the abnormal pathways of fatty acids such as linoleic acid, arachidonic acid, and histidine metabolism in ALI mice, restoring biomarkers such as L-carnosine and 1-methylhistidine levels (60).

## 4 Discussion

*Poria cocos*, as a medicinal and food dual-purpose herb, demonstrates vast application potential in modern pharmaceutical and food research. *Poria cocos* is documented to have diuretic, damp-dispersing, spleen-strengthening, stomach-soothing, and tranquilizing effects, but current research evidence has not deeply elucidated its mechanisms of action. Despite decades of research using modern experimental techniques, the latest studies still focus on the evaluation of Poria polysaccharides and crude extracts. Moreover, many compounds in *Poria cocos* have been isolated and identified, but research on the bioactivity of these compounds has not been fully conducted. The existing gap between compound composition and bioactivity research needs to be addressed. The author hopes that this review will provide valuable research ideas for the future development and application of *Poria cocos*, promoting the in-depth exploration of medicinal and food dual-purpose herbs.
